# Illuminating High-Affinity ATP Binding to the Sodium-Potassium Pump Using Solid-State NMR Spectroscopy

**DOI:** 10.3390/molecules30173609

**Published:** 2025-09-03

**Authors:** David A. Middleton

**Affiliations:** Department of Chemistry, Lancaster University, Bailrigg, Lancaster LA1 4YB, UK; d.middleton@lancaster.ac.uk; Tel.: +44-1524-594328

**Keywords:** solid-state NMR, Na,K-ATPase, torsional angle, ribose, density functional theory, REDOR, magic-angle spinning

## Abstract

Proteins that span cellular membranes represent around 30% of the proteome and over 50% of drug targets. A variety of synthetic and naturally-occurring small organic molecules interact with membrane proteins and up- and down-regulate protein function. The atomic details of these regulatory molecules offer important information about protein function and aid the discovery, refinement and optimization of new drugs. X-ray crystallography and cryo-electron microscopy (cryo-EM) are not always able to resolve the structures of small molecules in their physiological sites on membrane proteins, particularly if the molecules are unstable or are reactive enzyme substrates. Solid-state nuclear magnetic resonance (SSNMR) is a valuable technique for filling in missing details on the conformations, dynamics and binding environments of small molecules regulators of membrane proteins. SSNMR does not require diffracting crystals possessing long-range order and can be performed on proteins within their native membranes and with freeze-trapping to maintain sample stability. Here, work over the last two decades is described, in which SSNMR methods have been developed to report on interactions of the ATP substrate with the Na,K-ATPase (NKA), an ion-transporting enzyme that maintains cellular potential in all animals. It is shown how a combination of SSNMR measurements on membranous NKA preparations in the frozen and fluid states have provided unique information about the molecular conformation and local environment of ATP in the high-affinity nucleotide site. A combination of chemical shift analysis using density functional theory (DFT) calculations, dipolar coupling measurements using REDOR and measurements of the rates of proton spin diffusion is appraised collectively. The work described herein highlights the methods developed and challenges encountered, which have led to a detailed and unrivalled picture of ATP in its high-affinity binding site.

## 1. Introduction

Solid-state nuclear magnetic resonance (SSNMR) is a powerful technique in structural biology for elucidating the atomic details of biological molecules, materials and assemblies in disordered, microcrystalline or hydrated gel preparations [1]. Unlike diffraction techniques, SSNMR does not require the preparation of crystals possessing long-range order [2]. Since the 1990s, SSNMR practitioners have made considerable advances toward understanding the structures and dynamics of proteins that, historically, have been inaccessible to diffraction techniques. Notable examples are membrane-embedded protein targets of pharmaceutical drugs [3,4,5] and fibrous protein assemblies associated with Alzheimer’s disease and other protein misfolding disorders [6]. The protein analytes may be hydrated and not strictly solid, but restrictions in their motion hampers solution NMR analysis because of severe resonance line broadening. In contrast to solution NMR, SSNMR can exploit the advantages of anisotropic internal magnetic field interactions (chemical shielding, dipole–dipole, quadrupolar) in solids, whilst eliminating their undesirable effects. 

High-resolution NMR spectroscopy of solid materials and molecules is typically achieved with the assistance of magic-angle spinning (MAS), whereby the solid sample is mechanically rotated about an axis inclined at 54.7° relative to the applied magnetic field, *B*_0_. Recent advances in hardware technology have enabled MAS frequencies of above 100 kHz to be attained [7], facilitating the direct detection of protons and the development of a suite of NMR experiments analogous to those employed in biological solution-state NMR for decades [8]. NMR analyses of proteins typically require selective, sparse or uniform isotopic labelling of the biomolecule with combinations of ^2^H, ^15^N and ^13^C nuclei [9]. Rare nuclei with low sensitivity and/or long relaxation times (e.g., ^15^N, ^13^C) may also be observed directly with Hartmann–Hahn cross-polarization (CP) from the abundant proton reservoir, with the aid of high-power proton decoupling to eliminate heteronuclear dipolar interactions during signal acquisition [10]. This experimental combination is commonly referred to as CP-MAS SSNMR. 

At the time of writing (August 2025), the Protein Data Bank (PDB) contains the atomic coordinates of 185 protein and nucleic acid molecules and assemblies that have been determined by CP-MAS SSNMR. This number is very small compared to the numbers of structures determined by X-ray (~197,000), cryo-electron microscopy (cryo-EM) (~28,000) and solution-state NMR (~14,000). It should be noted, however, that SSNMR can provide focused information on biomolecular structure that is not accessible to other, more widely-used methods. For example, SSNMR is capable of focusing on the molecular conformations, dynamics and interaction sites of small organic entities bound to protein molecules and assemblies. Many physiological and pharmacological functions are performed by a wide array of small molecules, including accessory factors (e.g., glycan polysaccharides) that regulate the assembly of proteins into amyloid fibrils [11], and agonists, substrates, prosthetic groups and inhibitors of membrane protein drug targets [12,13]. Examples of the structural details of membrane protein ligands that have been resolved by SSNMR include the retinal chromophore in rhodopsins [14], anti-ulcer drugs bound to the gastric proton pump [15] and acetylcholine bound to the nicotinic acetylcholine receptor in native membranes [16]. Structurally-informative SSNMR measurements may be performed on functional protein–ligand complexes in native membranes that can be assayed for biological activity before and after the NMR measurements.

There are several practical considerations that should be addressed to enable the SSNMR detection of small molecules bound to proteins. The molecule of interest must usually contain appropriate NMR nuclei (e.g., ^13^C) at high levels of enrichment so that they can be detected unambiguously when bound to large proteins [9]. Isotope enrichment enhances sensitivity—only a few nanomoles of the small molecule may be present in the NMR sample—and helps to identify resonances from the molecule of interest in the presence of an overwhelming background NMR signal from naturally-abundant nuclei in the surrounding environment [17]. In favorable cases, the molecule of interest possesses functional groups containing NMR active isotopes such as ^31^P or ^19^F [15]. Sometimes the labelled molecule may be available commercially and there are a number of companies who supply “off-the-shelf” isotope labelled molecules. Otherwise, it is necessary to label the molecule in-house by chemical synthesis using commercially-available isotope labelled synthetic precursors [18,19]. In the case of membrane-embedded proteins, the sample must be prepared in a suitable lipid matrix, which may be the native membrane or a reconstitutive lipid bilayer [20]. Sample stability may also be an issue, particularly as measurement times may amount to days or weeks, and so sample freezing may be desirable during the measurements. It is important to perform temperature calibrations in advance and ensure that the gas supply for temperature control and sample spinning has a sufficiently low dew point. When possible, assays of protein function (e.g., ligand binding or enzyme activity) should be performed before and after the SSNMR measurements, to ensure that sample integrity is preserved. 

This article brings together our work over the past two decades using SSNMR to resolve the structural details of small molecules bound to the Na,K-ATPase (NKA), an essential membrane-embedded protein that occurs in all higher organisms. The examples presented illustrate the versatility of SSNMR for determining the structures, dynamics and interactions of membrane protein-bound small molecules, utilising chemical shifts and dipole–dipole interactions to report on interatomic distances and angles. 

## 2. The Na,K-ATPase

**Figure 1:**
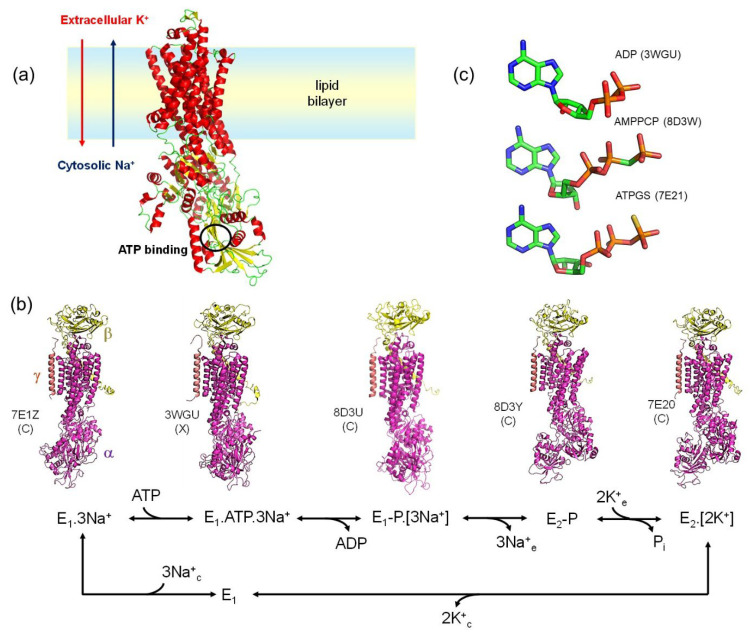
The Na,K-ATPase. (**a**) Structural overview. (**b**) Simplified catalytic cycle of NKA (α, β and γ subunits, colored magenta, yellow and orange, respectively), with crystal structures of the main intermediates. The E_1_.ATP.3Na^+^ conformation is the high-affinity nucleotide binding state, for which the crystal structures contain ADP or AMPPCP. Subscripts e and c denote extracellular and cytosolic, respectively. PDB IDs and method of structure determination (X = X-ray; C = cryo-EM) are shown. Intermediates binding ATP with low affinity (e.g., the E_2_-P.ATP and E_2_.ATP.[2K^+^] states) are not included. (**c**) Structures of ATP proxies bound to NKA (PDB ID in brackets).

NKA (EC 3.6.3.9) is a ubiquitous enzyme that is embedded in the plasma membranes of all animal cells (Figure 1a) [21]. It was discovered in the 1950s by Jens Christian Skou (one of the three 1997 Nobel Laureates in Chemistry) and was the first-identified member of a family of enzymes, the P-type ATPases, that catalyze ATP hydrolysis in two steps [22]. First, the γ-phosphate from ATP is transferred to the carboxyl group of Asp369, then the acyl-phosphate bond is hydrolyzed. NKA is a dimeric protein, with each monomer consisting of a catalytic a subunit (~110 kDa), glycosylated β subunit (~35 kDa) and a γ subunit (~10 kDa) containing the FXYD sequence. The cytoplasmic lobe of the a subunit contains the nucleotide-binding (N) domain, the phosphorylation (P) domain and the actuator (A) domain. The extracellular face of the a subunit presents a high-affinity binding site for a class of highly potent inhibitory molecules called cardiac glycosides, which include cardiotonic steroids derived from digitalis plants [23]. 

The primary function of NKA is to maintain cellular potentials by transporting 3 Na^+^ outward and 2 K^+^ inward against their electrochemical gradients at the expense of the energy of 1 ATP molecule. ATP hydrolysis occurs via the magnesium-catalyzed formation of a phosphorylated aspartic acid (Asp369) intermediate in the P domain [24]. Vectorial transport of Na^+^ and K^+^ occurs consecutively and is performed by two principal enzyme conformations called E_1_ and E_2_ (Figure 1b) [22,25]. The mechanism of energy transduction from the site of ATP hydrolysis site to the sites of ion transport involves transition between the E_1_ and E_2_ protein conformations. In the E_2_-conformation, which is induced by K^+^, the affinity for ATP is low [26]. Displacement of K^+^ from the transport sites by Na^+^ enables ATP and ADP binding with high affinity, a signature of the E_1_-conformation [27]. 

## 3. SSNMR Analysis of ATP Bound to Na,K-ATPase

A small but vital step in the NKA catalytic cycle is binding of ATP to the N domain of the enzyme in its E_1_ conformation. High-affinity ATP binding, induced by sodium ions, is essential to trigger the enzyme conformational change that releases 3 Na^+^ outside the cell. Knowledge of the molecular details of ATP binding is important, therefore, for a complete understanding of the enzyme’s function. 

Our first SSNMR study of ATP–NKA interactions was published in 2006, just before the first X-ray crystal structure of NKA was released (PDB ID 3KDP; 3.5 Å resolution) [28]. The crystal structure contained no information about bound ATP and so the SSNMR data offered the first clues about ATP interactions with the enzyme in an E_1_ conformation. Over the ensuing years, many more NKA structures have been determined by X-ray crystallography and cryo-electron microscopy (cryo-EM). There are now over 40 structures in the PDB of NKA in E_1_ and E_2_ conformations, including cryo-EM structures of the E_2_P state formed by ATP (7WYU, 7WYV; 3.4 Å) [29], the E_2_.2K^+^ state after addition of ATP (7Y46; 7.2 Å) [30], and E_1_ states with non-hydrolysable ATP-γ-S (ATPGS) (7E21; 2.9 Å) [31] or AMPPCP (in which the bridging oxygen of the terminal phosphodiester group is replaced by carbon) (8D3W; 3.5 Å) [32] (Figure 1b). X-ray structures include an Na^+^-bound pre-E_1_P state with bound ADP (3WGV; 2.8 A) [33] and an ADP-bound, Na^+^ occluded state (4HQJ, 8D3U; 4.3 A) [34]. The structures of the ATP analogues are quite variable in the conformations of the phosphate and ribose groups and in the relative orientations of the ribose and adenine rings. (Figure 1c). It is not clear which of them, if any, bears similarity with the conformation of authentic ATP in the high-affinity nucleotide site. SSNMR has filled in missing details about ATP binding that have not been addressed by the NKA structures to date. As will be shown, SSNMR has provided the only currently-available structural details for authentic ATP bound to the high-affinity site of NKA in the E_1_ conformation (i.e., most relevant to the catalytic cycle of the functional enzyme).

### 3.1. Detection of Nucleotide Binding to NKA in Native Membranes

**Figure 2:**
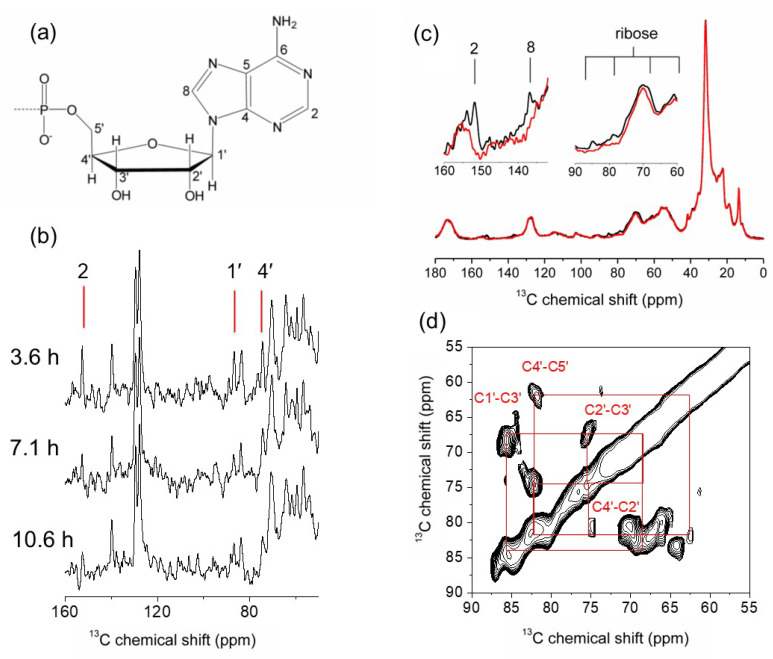
^13^C CP-MAS SSNMR analysis of membranous NKA after the addition of [U-^13^C,^15^N]ATP. (**a**) General chemical structure of adenine nucleotides, showing the ribose and adenine ring numbering convention. (**b**) Time-dependent spectra obtained by summation of 5 individual spectra of non-frozen membranes containing 4 mM [U-^13^C,^15^N]ATP. (**c**) Spectra obtained from frozen NKA membranes (at −25 °C) alone (red) and containing 160 mM [U-^13^C,^15^N]ATP (black). (**d**) 2D ^13^C-^13^C dipolar correlation spectrum of the sample in (**c**). Spectra were obtained at an applied magnetic field strength, *B*_0_, of 9.3 T. The MAS frequency was 4 kHz in (**b**), 5.1 kHz in (**c**) and 8 kHz in (**d**). No spinning sidebands appear in the spectral field of view. Adapted from [35].

Our early work demonstrated the feasibility of using ^13^C CP-MAS SSNMR to detect ATP binding to functional NKA in native membranes under non-frozen conditions [35]. Membranous NKA is prepared from pig kidney microsomal membranes [36] or rectal fin of the shark *Squalus acanthias* [37] and purified by differential centrifugation. Several milligrams of enzyme (about 45 mg/mL, equivalent to about 0.14 mM nucleotide sites) [35] can be obtained routinely. Preparations used for SSNMR have a specific NKA activity at 37 °C of about 1800 μmol ATP hydrolyzed/mg protein per hour, and the high-affinity nucleotide binding capacity is about 2.9 nmol/mg protein [38]. All our SSNMR studies benefited from the commercial availability of ATP (Figure 2a), in which all carbon and nitrogen sites are enriched with ^13^C and ^15^N, respectively ([U-^13^C,^15^N]ATP), thus avoiding the need for custom organic synthesis. An advantage of performing SSNMR measurements on non-frozen samples is that low MAS frequencies (3–5 kHz) are needed to suppress spinning sidebands, because intrinsic molecular dynamics in the fluid membranes contribute to the averaging of chemical shift anisotropy. Consequently, the centrifugal forces acting on the sample, which have the potential to affect membrane integrity and enzyme function, are relatively modest. Our assays of NKA activity before and after MAS at 4 kHz at 4 °C for 12 h confirmed a loss of around 10% activity. This is comparable to losses incurred at the same temperature without sample spinning (unpublished data) and confirms that centrifugal forces do not have a major impact on enzyme function. 

The ^13^C CP-MAS SSNMR detection of nucleotide binding to membranous NKA at 4 °C was assisted by Hartmann–Hahn cross-polarization (HHCP), which transfers magnetization from ^1^H to ^13^C, provided that heteronuclear dipolar interactions are not averaged by isotropic molecular reorientation (as occurs for ATP in solution). The practical consequence of the dependence of HHCP on non-zero ^1^H–^13^C dipolar coupling is that ^13^C resonances from ATP (or ADP) are only observed in the spectrum if the nucleotide is constrained within the NKA binding site [39]. A further consequence is that nucleotide binding kinetics (*k*_off_ in the microsecond range) are more favorable for sensitive NMR detection of the nucleotide than would be the case for stronger binding [40]. This is because the increased rate of exchange of ATP between free and bound states favors rapid signal build-up during cross-polarization compared to the rate of signal damping by rotating-frame (T_1_ρ) relaxation. 

NKA membrane preparations contain an enzyme activity that appears to be independent of NKA and which leads to hydrolysis of ATP, ADP and AMP [41]. Nucleotide hydrolysis is a significant problem for SSNMR measurements of ATP and ADP binding to NKA, because high protein concentrations and long acquisition periods (up to 5 h) are needed to achieve sufficient signal-to-noise. Accordingly, we found that signals from the nucleotide were lost within 1 h of measurement, and so several measures were taken to minimize ATP hydrolysis, extend measurement times and improve the signal-to-noise. The measures included (i) adding together spectra obtained from multiple samples obtained under identical conditions, (ii) removing residual Mg^2+^ from the sample by CDTA chelation and (iii) adding a high initial ATP concentration (4 mM). An alternative approach, not used here, is to regenerate ATP from ADP during the NMR measurements, via transfer of a phosphate from phosphoenolpyruvate (PEP) by a pyruvate kinase [42].

The ^13^C CP-MAS SSNMR spectrum of NKA membranes exhibits signature peaks from the bound nucleotide in the ranges 140–155 ppm and 80–90 ppm, together with peaks at 120–130 ppm and up-field of 80 ppm from naturally-abundant ^13^C from the lipids and protein in the sample (Figure 2b). The nucleotide signals persist for up to 10 h. However, using such a high initial ATP concentration runs the risk of introducing non-specific nucleotide binding [39], and so one cannot be confident that the observed signals fully and faithfully represent ATP binding to the high-affinity nucleotide site.

### 3.2. Experiments on Freeze-Trapped Nucleotide 

Much more highly detailed, and less ambiguous, information could be obtained by developing SSNMR procedures to measure the NKA-ATP complex under frozen conditions (−25 °C) after rapid freeze-trapping in liquid nitrogen [43]. With rapid freezing, a lower concentration of [U-^13^C,^15^N]ATP (160 µM) may be added to the membranous preparation without concern for nucleotide hydrolytic depletion over time. In the presence of Na^+^, around 130 µM (80–90%) of added nucleotide is bound to the high-affinity site of NKA. Although the lower ATP concentration requires much longer measurement times, on the order of several days or weeks to achieve the desired signal-to-noise, the nucleotide-NKA complex remains stable for this extended period provided freezing is maintained under a dry gas supply. Flash freezing in liquid nitrogen appears to have no effect upon NKA structural integrity (as implied by functional assays before and after freeze-thawing), but it is advisable to avoid repeated freeze-thawing cycles on the same sample to minimize cumulative detrimental effects on sample integrity. 

Figure 2c shows the ^13^C CP-MAS SSNMR spectrum of freeze-trapped membranous NKA alone and after addition of [U-^13^C,^15^N]ATP. With subtraction of background resonances from the natural-abundance ^13^C nuclei in the membrane lipids and protein, most of the ^13^C resonances for ATP can be detected. A 2D ^13^C–^13^C dipolar correlation SSNMR spectrum (Figure 2d) enables assignment of the ATP resonances and measurement of structurally-informative chemical shifts (see later). The observed ^13^C chemical shifts now predominantly reflect the bound state (80–90% of total ATP is locked in the binding site), whereas, in the non-frozen experiments, the chemical shifts are dynamically averaged by exchange of ATP between free and bound environments. Importantly, the improved sample stability and reduced dynamics opened the door to a range of more sophisticated SSNMR experiments exploring ATP conformation and binding environment.

### 3.3. Molecular Conformation of ATP in the High-Affinity Nucleotide Site

**Figure 3:**
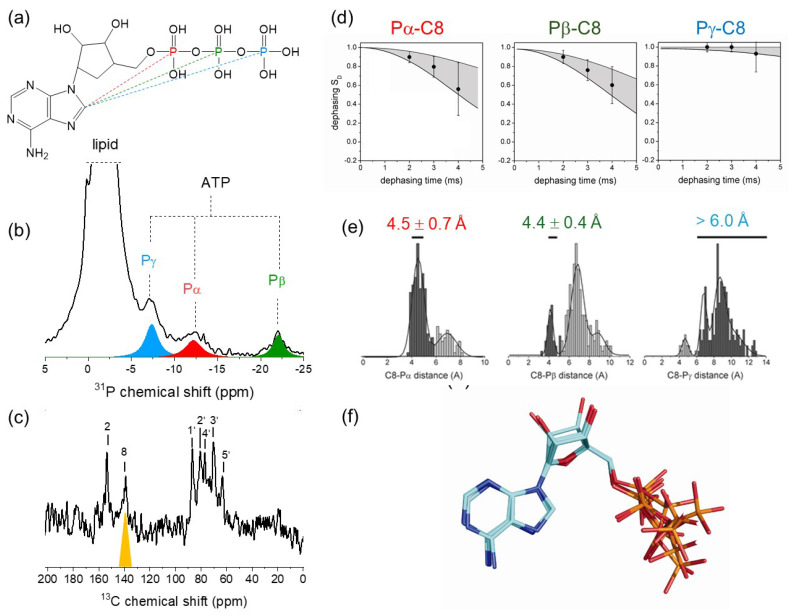
Molecular conformation of ATP. (**a**) Through-space C8-P distances in ATP measured by frequency selective ^31^P,^13^C-REDOR NMR. (**b**) ^31^P MAS SSNMR spectrum of membranous NKA preparation containing 160 mM [U-^13^C,^15^N]ATP, showing the 3 ATP phosphate resonances. (**c**) ^13^C CP-MAS SSNMR spectrum of the same sample, highlighting the resonance for C8. (**d**) REDOR dephasing and corresponding C8-P distance ranges. (**e**) Distribution of C8-P distances in >200 crystal structures of ATP-binding proteins. The dark shaded regions represent the distances measured for ATP bound to NKA. (**f**) Statistically-constrained distribution of conformations consistent with the SSNMR data and known ATP conformations. Adapted from [3].

The development of rapid freeze-trapping enabled us to determine the conformation of ATP when locked within the NKA nucleotide site, using the rotational-echo double-resonance (REDOR) SSNMR experiment [3]. This experiment reintroduces heteronuclear dipolar couplings under MAS conditions, and a frequency-selective DANTE-^31^P(^13^C)-REDOR version of the experiment [44] was used to detect the rate of dipolar dephasing of ^31^P coherence from ATP, by selectively reintroducing ^31^P–^13^C dipolar couplings between C8 of the adenine ring and Pα, Pβ and Pγ. The de-phased NMR signal, *S*_D_, is a function of the strength of the individual ^31^P–^13^C dipole-dipole interactions, which, in turn, are inversely proportional to the cube of the internuclear distance (Figure 3a). Estimates of the three phosphorus-carbon distances for ATP can, in principle, constrain the possible molecular conformations of the bound nucleotide. 

*S*_D_ is measured by comparing the peak intensities for Pα, Pβ and Pγ before and after selective recoupling of C8 (Figure 3b). The measured ^31^P-^13^C distances were confined to relatively narrow ranges for each carbon–phosphorus pair, but they only weakly constrained the allowed conformational space of bound ATP. To improve the constraining power, the SSNMR measurements combined with a statistical analysis of the conformational preferences of ATP, ADP and AMP ligands from 437 protein structures in the PDB (Figure 3c). By doing so, the NKA-bound ATP conformation could be constrained to five closely-related clusters with similar relative orientations of the adenine and ribose rings (Figure 3d).

### 3.4. Further Conformational Restraints

**Figure 4:**
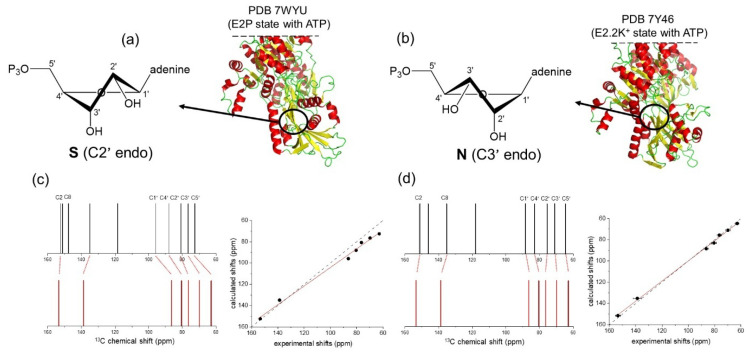
DFT analysis of ATP ^13^C chemical shifts. (**a**) Structure of the E_2_P state of NKA, showing the C2′ endo conformation of the ATP ribose ring. (**b**) Structure of the E_2_.2K^+^ state of NKA, showing the C3′ endo conformation of the ATP ribose ring. (**c**) Comparison of the experimental ^13^C chemical shifts of ATP bound to the high-affinity site of NKA in the E_1_ conformation (top, black) and calculated shifts for the ribose ring in the C2’ endo conformation (bottom, red). (**d**) Better agreement is seen between the experimental shifts and calculated shifts for the ribose ring in the C3’ endo conformation. Adapted from [45].

Further restraints on the ribose ring conformation of ATP could be applied by analyzing the ^13^C isotropic chemical shifts of the bound nucleotide. Pseudo-rotation of ribose rings in nucleotides results in two major conformations, referred to as the S (or C2^′^-endo) and N (or C3^′^-endo) forms (Figure 4). Structures of NKA in E_2_ states, in which ATP is bound in the low affinity site, give conflicting information on the ribose conformation: in one structure the ribose ring adopts the S form and in the other structure ATP adopts the N-form (Figure 4). There is no information on the ribose ring structure in high-affinity E_1_ conformations. We compared experimental ^13^C chemical shift values with shift values calculated using density functional theory (DFT) for ATP in the S and N forms [45]. The comparison revealed a close match with the N-form, providing the first compelling evidence for the ATP ribose ring conformation when bound to the high-affinity site.

**Figure 5:**
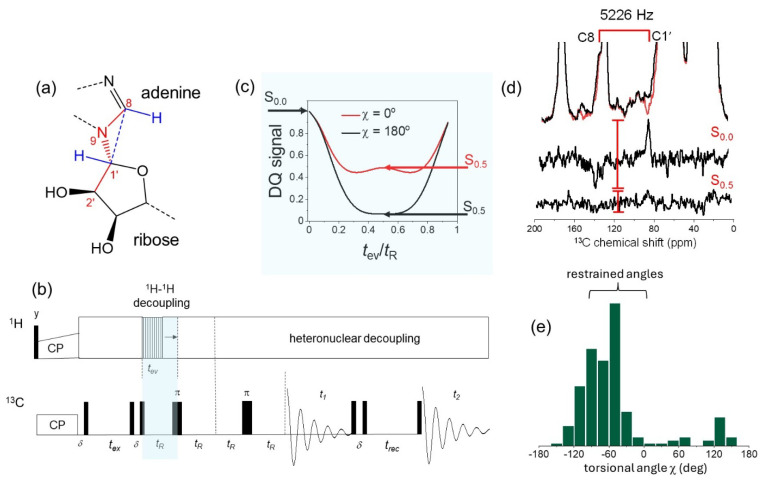
Analysis of the adenine–ribose ring orientation in NKA-bound ATP. (**a**) Definition of torsional angle c for ATP, and its relationship with the C8 – H and C1’ – H bond orientations. (**b**) Selective DQ HCCH SSNMR pulse sequence, highlighting the variable DQ evolution period, *t*_ev_ (blue), where *t*_ev_ is less than or equal to one rotor period, t_R_. (**c**) Dependence of the DQ signal intensity, *S*, as a function of *t*_ev_ when c = 0° (red) and c = 180° (black). (**d**) Selective DQ HCCH SSNMR spectra showing the peak intensities at *t*_ev_/*t*_R_ = 0 (S_0.0_) and *t*_ev_/*t*_R_ = 0.5 (S_0.5_). (**e**) Distribution of angle c, measured from coordinates ATP bound to 272 diverse and unrelated proteins from the PDB. The restrained range of angles consistent with the SSNMR data is highlighted. Adapted from [45].

A further structural variable is the orientation of the ribose ring relative to the adenine base, which is defined by torsional angle χ (C8–N9–C1′–C2′) (Figure 5a). The relative orientation in bound ATP was obtained with a frequency-selective version of the double quantum (DQ) HCCH SSNMR experiment to determine the relative orientations of adenine C8–H and ribose C1’–H bonds (Figure 5b,c) [46,47]. Restraints on χ are obtained from the peak intensities for C8 and C1’ after selective excitation of DQ coherence at *n* = 1 rotational resonance (i.e., the MAS frequency is set to 5226 Hz, the difference in resonance frequencies of C8 and C1’) (Figure 5d). Analysis of ATP bound to structurally-defined proteins in the PDB indicates that angle c can take a range of possible values between −180° and +180° (Figure 5e). The DQ HCCH spectra restrict the range of angles to between 0° and −130°, adding a further restraint to the overall molecular conformation of ATP in the high-affinity nucleotide site.

### 3.5. Exploring the Binding Environment of ATP

SSNMR methods can also be used to gain insight into the surroundings of ATP in the high-affinity nucleotide site, which have remained elusive to X-ray crystallography and cryo-EM. 

**Figure 6:**
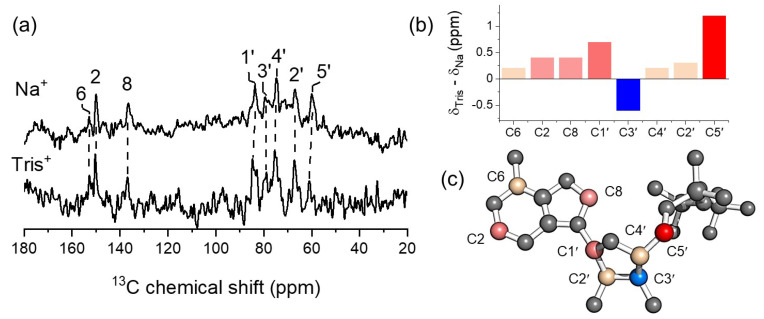
SSNMR reveals differences in high-affinity ATP binding to NKA induced by Na^+^ and Tris^+^. (**a**) ^13^C CP-MAS SSNMR spectra. (**b**) Chemical shift differences for bound ATP in the presence of the two cations. (**c**) The chemical shift differences mapped onto the structure of ATP.

As stated earlier, high-affinity ATP binding is induced by the presence of Na^+^, but other mono-cations such as Tris^+^ ((HOCH_2_)_3_CNH_3_^+^) are equally efficient promoters of nucleotide binding. The ^13^C chemical shifts for bound [U-^13^C,^15^N]ATP with NKA purified from pig kidney membranes reveals subtle differences in the nucleotide interactions within the nucleotide site (Figure 6a–c), depending on whether Na^+^ or Tris^+^ is used to induce binding [48]. The chemical shift differences are greatest for ribose atoms C1′, C3’ and C5′ and reflect variances in the nucleotide binding pocket and/or the conformation of the ribose ring. According to DFT calculations, the chemical shift differences are not sufficient to explain a ribose conformational change from the N-form to the S-form, but could represent a puckering of the ring in the presence of Tris^+^. 

**Figure 7:**
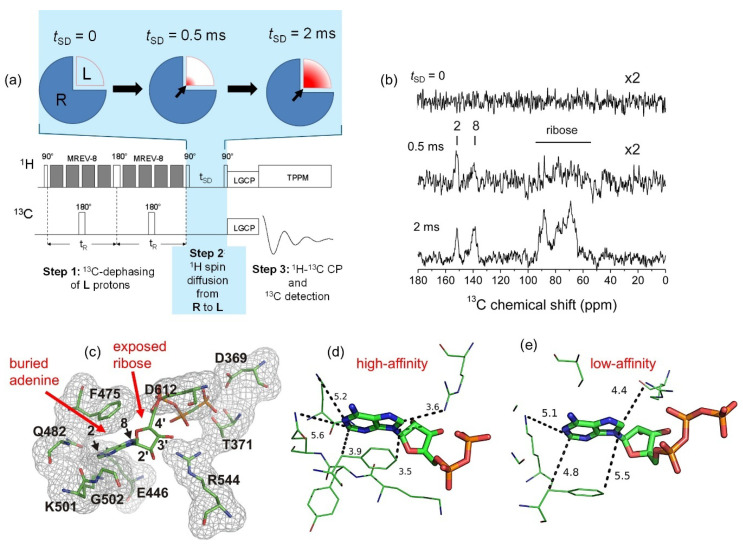
Probing contacts of ATP with the nucleotide site. (**a**) The ^13^C-detected PSD SSNMR pulse scheme. The MREV-8 (multiple-pulse effective-field resonance-8) sequence suppresses ^1^H–^1^H dipolar couplings during the evolution of proton spins and the Lee–Goldburg cross-polarization (LG-CP) step suppresses ^1^H–^1^H couplings during ^1^H–^13^C magnetization transfer. (**b**) Spectra obtained at different proton spin diffusion periods, *t*_SD_. (**c**) Homology model of ATP in the high-affinity nucleotide site of NKA, based on the SERCA2a structural template. (**d**) Crystal structure of ADP binding to NKA in a high-affinity (E_1_.ATP.3Na^+^) state. The dashed lines indicate the closest distances (labelled in Angstroms) between ATP and residues in the binding site. (**e**) Crystal structure of ATP binding to NKA in a low affinity (E_2_.2K^+^) state.

Contacts between [U-^13^C,^15^N]ATP and the NKA nucleotide site were investigated further using a ^13^C-detected proton spin diffusion (PSD) experiment [43]. The aim of this experiment is to identify the nucleotide carbon sites in proximity to the binding pocket, by exploiting a ^13^C and ^15^N-labelled ligand molecule, **L** ([U-^13^C,^15^N]ATP) bound to an unlabeled receptor, **R** (NKA). The general strategy is illustrated in Figure 7a. In Step 1 the ^1^H magnetization for bound **L** is silenced by dephasing, by reintroducing proton dipolar interactions with ^13^C (and/or ^15^N) spins. The ^1^H magnetization of **R** remains largely unperturbed because the vast majority of the NKA protons are bonded to ^12^C at natural abundance. 

In Step 2, residual proton magnetization from **R** is transferred to **L** protons by spin diffusion over a variable period, *t*_SD_. The rate of spin diffusion is highest for **L** and **R** protons that are closest together. In Step 3, the ^1^H magnetization is transferred to ^13^C via HHCP and the ^13^C signal is detected. With judicious selection of *t*_SD_, it is possible to detect selectively the carbon sites of **L** in closest contact with the binding site of **S**. For [U-^13^C,^15^N]ATP bound to NKA, no spin diffusion occurs when *t*_SD_ is zero (Figure 7b, top) and when *t*_SD_ is relatively long (2 ms) all nucleotide ^13^C resonances are detected indiscriminately (Figure 7b, bottom). At an intermediate spin diffusion period (*t*_SD_ = 0.5 ms), the adenine ^13^C resonances (C2 and C8) are more pronounced than the ribose resonances (Figure 7b, middle), indicating that the adenine ring is likely to be buried within a binding pocket, whereas the ribose ring is relatively exposed. When the original work was published, no crystal structures of NKA with bound ATP or ATP analogues were available to verify the SSNMR conclusions. Instead, the conclusions were supported by homology modelling the ATP–NKA complex, using as a structural template the sarco(endo)plasmic reticulum Ca^2+^-ATPase (SERCA2) with bound ATP analogue AMPPCP (Figure 7c). The conclusions can now be reconciled with recent published structures of NKA with ATP in the low-affinity site (E_2_.2K^+^ state; 7Y46) and of NKA with ADP in a high-affinity NKA form (E_1_.ATP.3Na^+^; 3WGU) (Figure 7d). The high-affinity structure clearly shows that more amino acid residues are closer to the ATP adenine ring than are residues in the low-affinity structure, whereas the ribose ring remains relatively exposed in both structures.

**Figure 8:**
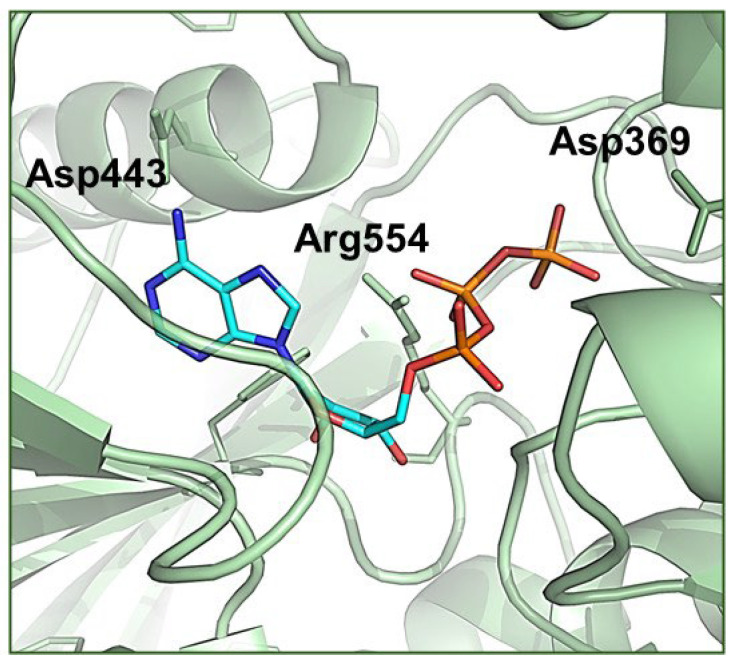
Model of ATP in the high-affinity nucleotide site of NKA in the E_1_ conformation, from the combined SSNMR restraints and data described herein.

## 4. Conclusions and Outlook

This summary of our work over the past two decades illustrates some of the SSNMR methodology and other experimental procedures that can be used to determine the molecular conformations and binding environment of small organic molecules when bound to membrane proteins. In this context, SSNMR has a number of distinct advantages over X-ray diffraction and cryo-EM. Diffracting crystals are not required, functional proteins can be studied in their native membranes, and unstable or labile molecules can be freeze-trapped for measurements over many days or weeks. Here, bringing together REDOR distance information, ^13^C chemical shift restraints and spin-diffusion measurements obtained over several years, a refined model of ATP bound to the high-affinity nucleotide site of NKA is presented (Figure 8). SSNMR is currently the only technique that has provided this information. 

In common with other techniques, SSNMR has costs, technical challenges and other limitations that must be considered against the value of the information sought. Magnetic resonance is inherently insensitive owing to the near parity of nuclear spin state populations and, even with persistent magnetic fields of over 20 Tesla, SSNMR typically requires milligram quantities of protein to achieve acceptable signal-to-noise. High amounts of NKA can be purified from animal (shark and pig) tissue, but in other cases cell lines overexpressing the protein of interest are needed. For membrane protein analysis, although crystals are not required, suitable lipid bilayer preparations supporting functionally and structurally intact proteins must be developed. Further, isotope labelling of small molecule ligands is often labor intensive and/or expensive. Despite advances in direct proton detection by SSNMR at high MAS rates, isotope labelling of proteins and their bound ligands is still needed for resolution enhancement and signal filtration. The increasing availability of affordable fluorine-labelled molecules offers some mitigation against these challenges. Having a high gyromagnetic ratio, the ^19^F nucleus is suitable for measuring structurally-informative dipolar couplings probing internuclear distances of over 10 Å. Fluorinated ATP analogues are commercially available and have been exploited in solution-state NMR. 

Structural characterization of NKA has reached maturity and it is unlikely that SSNMR will provide many more useful insights into nucleotide binding to this enzyme, particularly as advances in cryo-EM continue to gather momentum. Looking forward, six P-type ATPase families, termed P1 to P6 have been identified, with members found in all living organisms, and little or no structural information is available for many of these [49]. With suitable over-expression systems, SSNMR can play an important role in determining the similarities and differences in nucleotide binding to these enzymes, and their coupling with metal transportation. Regarding the latter function, it is important to mention that SSNMR can monitor the local chemical environment of metal cations associated with P-type ATPases. NMR-active nuclei include the cations ^87^Rb^+^, ^133^Cs^+^, and ^205^Tl^+^, which can act as K^+^-substitutes in the E_2_-occluded NKA state [50,51]. SSNMR can report different chemical environments and stoichiometries of the cations according to their separation in the spectrum and resonance intensities. If paramagnetic cations are incorporated into P-type ATPases, it is also possible to measure distances between metal and organic ligand binding sites within the protein by observing dipolar broadening [52]. 

## Data Availability

No new data were created in this study. Data sharing is not applicable to this article.
